# The Effect of Different Timings of Delayed Cord Clamping of Term Infants on Maternal and Newborn Outcomes in Normal Vaginal Deliveries

**DOI:** 10.7759/cureus.17169

**Published:** 2021-08-14

**Authors:** Divya Katariya, Dharitri Swain, Sweta Singh, Arti Satapathy

**Affiliations:** 1 College of Nursing, All India Institute of Medical Sciences, Bhubaneswar, Bhubaneswar, IND; 2 Obstetrics and Gynaecology Nursing, All India Institute of Medical Sciences, Bhubaneswar, Bhubaneswar, IND; 3 Obstetrics and Gynaecology, All India Institute of Medical Sciences, Bhubaneswar, Bhubaneswar, IND; 4 Obstetrics and Gynaecology, District Hospital Khurda, Khurda, IND

**Keywords:** bilirubin level, birth outcomes, delayed cord clamping, hemoglobin level, maternal blood loss

## Abstract

Background and objective

Delayed cord clamping (DCC) has proven to be an ideal approach to reduce iron deficiency anemia; however, different timings of DCC relative to the birth outcome lead to conflicting results. The present study was conducted to determine the effects of different timings of DCC on the maternal and neonatal outcomes in normal vaginal deliveries at term.

Methods

This was an interventional study on neonates born at term without complications to mothers with uneventful pregnancies in the labor unit of a district hospital in Odisha, India. A total of 147 women were randomized to three intervention groups: DCC at one minute, DCC at two minutes, and DCC at three minutes. Hemoglobin and bilirubin levels, maternal blood loss, the timing of the third stage of labor, oxytocin use, and birth weight of the neonates were measured as the outcomes of different timings of DCC.

Results

At 24-48 hours of age, hemoglobin and bilirubin levels of the neonates were significantly higher with DCC at three minutes compared to DCC at one and two minutes. However, there were no significant differences among the three groups in terms of the need for phototherapy. The duration of the third stage of labor was significantly longer with DCC at three minutes. Maternal blood loss, oxytocin use, and birth weight of the neonates were not significantly associated with the timing of DCC.

Conclusion

Based on our findings, waiting to clamp the umbilical cord until three minutes can effectively reduce the incidence of iron deficiency anemia in newborns.

## Introduction

Anemia is a major health concern worldwide, and it can lead to morbidity and mortality in young children. Globally, around 1.6 billion people are anemic, and the majority of the affected individuals are young school-aged children [1,2]. In Asia, India is severely affected by this condition, with around 90 million people suffering from anemia, of which 70% are children in the age group of 6-59 months [1]. Odisha is one among the eight Empowered Action Group states of India, where 64% of the children in the age group of 6-59 months are anemic [1]. Iron deficiency anemia represents a serious medical issue among children in low socioeconomic regions, and anemia prevention is crucial to promote the well-being of children [3]. Delayed cord clamping (DCC) has proven to be an effective approach to overcome this issue; however, variations in the timing of DCC have limited its application in the clinical setting [4-7].

Furthermore, many obstetricians and midwives are not aware of the precise timing or benefits related to DCC. Even today, there are certain ambiguities related to not only the timing but also the benefits versus risks of DCC [5]. Despite the availability of established guidelines by the World Health Organization (WHO), there are still gaps that need to be addressed in the practice of DCC in the clinical setting [2,8,9].

To this end, the present study was performed to determine the effects of different timings of DCC on the neonatal outcomes of hemoglobin and bilirubin levels and birth weight at 24-48 hours as well as the maternal outcomes of blood loss, length of the third stage of labor, and oxytocin use in normal vaginal deliveries at term.

## Materials and methods

This was a hospital-based, interventional study conducted from September to December 2019 in the labor unit of the District Headquarters Hospital, Khurda, Odisha, located in rural southeastern India. This district hospital has six delivery tables, and approximately 1236 vaginal deliveries were performed during the four-month study period. The study protocol was approved by the Institutional Ethics Committee (Ref No: IEC/AIIMS BBSR/Nursing/2018-19/13), and due permission was obtained from the local authorities of the study setting. Women presenting with a singleton gestation who were ≥18 years of age, were 37-42 weeks into pregnancy, and had been admitted for a vaginal birth without any complications were included in the study. Women with high-risk pregnancies or requiring urgent resuscitation, whose neonates presented with respiratory distress syndrome, and whose neonates did not cry immediately after birth were excluded. A consecutive sampling technique was used to recruit 147 participants meeting the inclusion criteria. After ensuring the confidentiality of the information provided by each participant, the study participants were told about the purpose of the study, and informed written consent was obtained from them. Finally, 132 cases were analyzed since 15 samples were lost due to non-compliance with sampling criteria, accounting for less than 10% of the entire sample.

**Figure 1:**
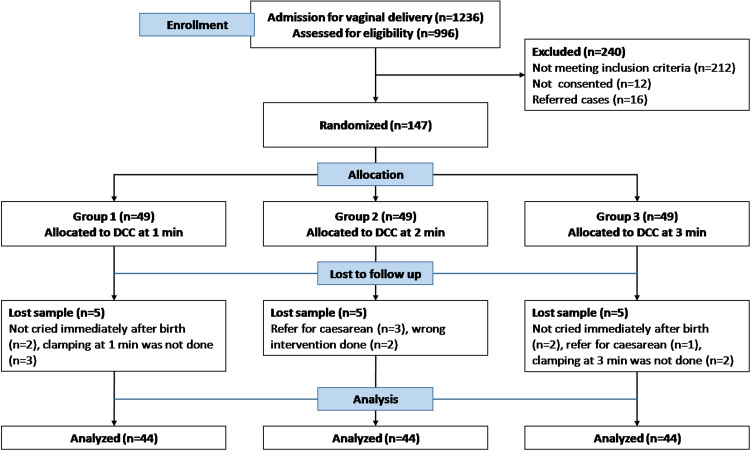
Flow diagram detailing the study design

Figure 1 presents the flow diagram illustrating participant recruitment. The participants were randomly assigned to three parallel groups at a 1:1:1 ratio based on the following criteria: DCC at one minute, DCC at two minutes, and DCC at three minutes. Sociodemographic tools and standardized biophysical instruments were used to measure clinical parameters. Sociodemographic parameters included the demographics and obstetrics history of the participants. Hemoglobin level was measured using a hemoglobinometer validated by the All India Institute of Medical Sciences, New Delhi. Bilirubin level was measured using a transcutaneous bilirubin meter, and when the value exceeded 12.0 mg/dL, total serum bilirubin was measured. A postpartum hemorrhage measuring sheet was used to measure maternal blood loss. It is a triangular drape (V-shaped), also known as the gallop sheet or BRASSS-V Drape [1]. The broader part of the sheet is placed under the mother’s buttocks, and the narrow V-shaped apex has a measurement hung below the buttocks. After discarding the placenta, only blood collected in the gallop sheet was measured to determine blood loss. A stopwatch was used to calculate the length of the third stage of labor and the timing of DCC. Oxytocin use was observed by an investigator directly at the time of the administration.

SPSS Statistics version 20 (IBM, Armonk, NY) was used to analyze the data. The baseline data obtained were expressed in frequency (%) and mean and standard deviation. The difference between the interventions and outcome variables was calculated to determine the significant improvements in the outcome measures using one-way analysis of variance (ANOVA) for normally distributed continuous data. The Chi-square test was used to analyze the associations in categorical data.

## Results

A total of 147 participants met the inclusion criteria, of which 15 were excluded due to non-compliance with sampling criteria, and 132 participants were included in the final analysis. The mean age of the parturient mothers was 25.71 ±4.6 years. The majority of the participants were multigravida women [74 (56%)] and 58 (44%) were primipara mothers. Regarding the gender of neonates, 71 (53.8%) newborns were male and 61 (46.2%) were female. In the DCC at one minute group, 54.4% of newborns were male and 45.5% were female. In the DCC at two minutes group, 50% of newborns each were male and female. In the DCC at three minutes group, 56.8% of newborns were male and 43.2% were female. The mean hemoglobin level at birth was 15 ±2.5, 15 ±2.4, and 14 ±2.2 g/dL in the DCC at one minute, DCC at two minutes, and DCC at three minutes groups, respectively (Table 1).

**Table 1:** Comparison of sociodemographic and clinical characteristics of subjects across the groups DCC: delayed cord clamping; SD: standard deviation

Variables	Timing of delayed cord clamping		
	Group 1 (DCC at 1 minute) (n_1_=44)	Group 2 (DCC at 2 minutes) (n_2_=44)	Group 3 (DCC at 3 minutes) (n_3_=44)
Age of mother (mean ±SD)	26.64 ±4.53	25.32 ±4.07	27.18 ±5.12
Parity, n (%)			
Primipara	18 (41%)	25 (57%)	15 (34%)
Multigravida	26 (59%)	19 (43%)	29 (66%)
Religion, n (%)			
Hindu	39 (88.6%)	43 (97.7%)	44 (100%)
Muslim	05 (11.4%)	01 (2.3%)	-
Gender of the baby, n (%)			
Male	24 (54.5%)	22 (50%)	25 (56.8%)
Female	20 (45.5%)	22 (50%)	19 (43.2%)
Maternal hemoglobin (gm/dl)			
Mean ±SD	15 ±2.5	15 ±2.4	14 ±2.2

Table 2 presents a comparison of birth outcomes among the three groups. At 24-48 hours of age, the mean hemoglobin level in the DCC at three minutes group (16.63 ±1.33 g/dL) was significantly higher than that in the other two groups (16.26 ±2.03 g/dL in DCC at two minutes and 15.30 ±1.93 g/dL in DCC at one minute). At 24-48 hours of age, the mean bilirubin level in the DCC at three minutes group (10.24 ±2.20 mg/dL) was significantly higher than that in the other two groups (9.29 ±1.24 mg/dL in DCC at one minute and 8.73 ±1.50 mg/dL in DCC at two minutes). The neonates in the DCC at three minutes group developed jaundice more frequently than those in the other two groups. However, there were no significant differences among the three groups in terms of the need for phototherapy. The mean birth weight was higher in the DCC at one minute group than in the other two groups, although the difference was not statistically significant (p=0.26).

Regarding maternal outcomes, the mean duration of the third stage of labor was significantly longer in the DCC at two minutes (8.65 ±2.98 minutes) group than in the other two groups (p=0.024). Mean oxytocin dose in the DCC at three minutes group (10.00 ±0.00 IU) was lower than that in the other two groups (10.23 ±1.50 IU in DCC at one minute and 10.45 ±2.10 in DCC at two minutes), although the difference was not statistically significant (p=0.36). There were no significant differences in mean maternal blood loss among the three groups.

**Table 2:** Comparison of birth outcomes across the groups χ2: Chi-square level of significance at p<0.05*; F: one-way ANOVA level of significance at p<0.05* ANOVA: analysis of variance; DCC: delayed cord clamping; SD: standard deviation

Variables	Timing of delayed cord clamping				Test statistics, p-value
	Group 1 (DCC at 1 minute) (n_1_=44)	Group 2 (DCC at 2 minutes) (n_2_=44)		Group 3 (DCC at 3 minutes) (n_3_=44)	
Comparison of newborn outcomes					
Hemoglobin (gm/dl)					
Mean ±SD	15.30 ±1.93		16.26 ±2.03	16.63 ±1.33	F=6.438, p=0.00*
Bilirubin (mg/dl)					
Mean ±SD	9.29 ±1.24		8.73 ±1.50	10.24 ±2.20	F=8.804, p=0.002*
Need of phototherapy, n (%)					
No	43 (97.7%)		42 (95.5%)	40 (90.9%)	x^2^=2.047, p=0.359
Yes	01 (2.3%)		02 (4.5%)	04 (9.09%)	
Birth weight (kg)					
Mean ±SD	2.85 ±0.42		2.72 ±0.42	2.82 ±0.33	F=1.310, p=0.273
Comparison of maternal outcomes					
Oxytocin (IU)					
Mean ±SD	10.23 ±1.50		10.45 ±2.10	10.00 ±0.00	F=1.016, p=0.365
Time of the third stage of labor (minutes)					
Mean ±SD	7.64 ±2.16		8.65 ±2.98	7.29 ±1.89	F=3.856, p=0.024*
Blood loss (ml)					
Mean ±SD	333.18 ±62.535		341.86 ±97.309	346.77 ±50.351	F=0.393, p=0.676

## Discussion

Compared with early cord clamping (ECC), DCC can effectively increase hemoglobin and serum ferritin levels and prevent anemia among newborns [5,10-14]. The present study confirmed the benefit of DCC among newborns, as evidenced by the significantly increased hemoglobin levels at 24-48 hours of age with DCC at three minutes compared to that with DCC at one or two minutes. Our finding is well supported by previous evidence that the mean hematocrit was higher in DCC at 180 seconds, followed by DCC at 120 and 60 seconds [15]. Likewise, in a previous study, the mean hematocrit was higher with DCC at three minutes than with DCC at two minutes and ECC within 15 seconds, and more cases of anemia were identified following ECC than DCC at six months of life [16]. In contrast, in another study, there were no significant differences in hemoglobin level at 24-48 hours of age between DCC at two and three minutes [9].

In a systematic review, DCC was reported to increase the mean hemoglobin level by up to 2-3 g/dL, which could be sustained up to six months of age [17]. In the present study, the mean hemoglobin level in the DCC at three minutes group was higher by 1.3 g/dL than that in the DCC at one minute group.

However, DCC is associated with a few potential complications, including hyperbilirubinemia and polycythemia. In many trials, more hyperbilirubinemia cases were identified following DCC than ECC [8,13,17,18]. Consistently, in the present study, bilirubin levels in the DCC at three minutes group were higher than those in the DCC at one and two minutes groups. However, there were no significant differences among the three groups in terms of the need for phototherapy, and all neonates requiring phototherapy were well managed without any adverse complications. In other studies, there were no significant differences in terms of the requirement of phototherapy to treat jaundice and the incidence of hyperbilirubinemia between DCC and ECC [9,12,16,19,20].

In the present study, there was no significant increase in birth weight with DCC. This finding is consistent with the observation in a randomized control trial [20]. However, another similar trial reported increased birth weight with DCC at three minutes compared to that with DCC at two minutes [9].

Postpartum hemorrhage is one of the leading causes of maternal death globally. Maternal blood loss can occur due to various reasons. In a previous study, DCC was found to be a reason underlying the increased incidence of postpartum hemorrhage [2]; however, much evidence indicates that DCC is not associated with maternal outcomes in terms of postpartum hemorrhage, oxytocin use, and duration of the third stage of labor [9,12,21-24]. In the present study, while DCC at one, two, and three minutes was not associated with maternal blood loss and oxytocin use, DCC at two minutes prolonged the duration of the third stage of labor.

Finally, some limitations of the present study related to its design must be considered. The study was not designed or registered as a clinical trial due to practical applicability. Blinding of the study was not possible with this design, as the intervention had to be performed in a time-bound manner; however, all attempts to blind the study were made when possible to reduce bias. Finally, 15 samples were lost due to non-compliance with sampling requirements, but the study's outcome was unaffected because the attrition rate was less than 10%.

## Conclusions

Based on our findings, DCC at three minutes can effectively increase hemoglobin levels in newborns, thereby reducing the incidence of iron deficiency anemia in the later stages of life; however, DCC can also increase the risk of neonatal jaundice, albeit without any adverse effects or further complications. There were no significant differences in DCC at one, two, and three minutes in terms of maternal outcomes such as postpartum hemorrhage and oxytocin use labor induction, but DCC at two minutes prolonged the duration of the third stage of labor. Overall, waiting to clamp the umbilical cord until three minutes can improve birth outcomes in terms of anemia.
